# Management strategies and outcomes predictors of interstitial lung disease exacerbation admitted to an intensive care setting: A narrative review

**DOI:** 10.2478/jccm-2025-0013

**Published:** 2025-04-30

**Authors:** Ans Alamami

**Affiliations:** Department of Critical Care Medicine, Hamad General Hospital, Doha, Qatar

**Keywords:** ILD, diffuse parenchymal lung disease, ICU

## Abstract

**Background:**

Interstitial lung disease (ILD) is a cluster of diseases that affect the lungs, characterized by different degrees of inflammation and fibrosis within the parenchyma. In the intensive care unit (ICU), ILD poses substantial challenges because of its complicated nature and high morbidity and mortality rates in severe cases. ILD pathophysiology frequently entails persistent inflammation that results in fibrosis, disrupting the typical structure and function of the lung. Patients with ILD frequently experience dyspnea, non-productive cough, and tiredness. In the ICU setting, these symptoms may worsen and lead to signs of acute respiratory failure with significantly impaired gas physiology.

**Methodology:**

A systematic search was conducted in reputable databases, including PubMed, Google Scholar, and Embase. To ensure a comprehensive search, a combination of keywords such as “interstitial lung disease,” “intensive care,” and “outcomes” was used. Studies published within the last ten years reporting on the outcomes of ILD patients admitted to intensive care included.

**Result:**

Effective management of ILD in an ICU setting is challenging and requires a comprehensive approach to address the triggering factor and providing respiratory support, Hypoxemia severity is a critical predictor of mortality, with lower PaO_2_/FiO_2_ ratios during the first three days of ICU admission associated with increased mortality rates. The need for mechanical ventilation, particularly invasive mechanical ventilation (IMV), is a significant predictor of poor outcomes in ILD patients. Additionally, higher positive end-expiratory pressure (PEEP) settings, and severity of illness scores, such as the Acute Physiology and Chronic Health Evaluation (APACHE) score, are also linked to increased mortality. Other poor prognostic factors include the presence of shock and pulmonary fibrosis on computed tomography (CT) images. Among the various types of ILDs, idiopathic pulmonary fibrosis (IPF) is associated with the highest mortality rate. Furthermore, a high ventilatory ratio (VR) within 24 hours after intubation independently predicts ICU mortality.

**Conclusion:**

This literature review points out outcome predictors of interstitial lung disease in intensive care units, which are mainly hypoxemia, the severity of the illness, invasive ventilation, the presence of shock, and the extent of fibrosis on CT Images.

## Introduction

Interstitial lung disease (ILD) is a cluster of diseases that affect the lungs, characterized by varying degrees of inflammation and fibrosis within the parenchyma. In the intensive care unit (ICU), ILD poses significant challenges due to its complex nature and high morbidity and mortality rates. This literature review aims to provide an understanding of the factors that determine outcomes for patients with ILD admitted to ICUs. The pathophysiology of ILD often involves persistent inflammation leading to fibrosis, which disrupts the normal structure and function of the lung. The gradual accumulation of fibrosis results in a marked decline in lung function and impaired gas exchange, ultimately leading to hypoxemia and respiratory failure. This process is believed to begin with an acute injurious event to the lung parenchyma, triggering persistent inflammation in the interstitial tissue, fibroblast activation and proliferation, and eventually pulmonary fibrosis and lung tissue destruction. Additionally, the structure of the airways may also be affected to varying degrees.

ILD typically presents with gradually developing respiratory symptoms, though it can occasionally manifest with sudden and rapidly worsening onset, as seen in cryptogenic organizing pneumonia (COP) or acute interstitial pneumonia (AIP), which resembles the clinical picture of acute respiratory distress syndrome (ARDS). Epidemiological data indicate that ILD affects approximately 30 individuals per 100,000 annually. According to a 1994 study from Bernalillo County [1], men are more frequently affected by ILD than women, with annual incidence rates estimated at 31.5 per 100,000 for males and 26.1 per 100,000 for females [1]. Age and occupational exposures also significantly influence the development of ILD. Connective tissue diseases and other rheumatologic conditions are more common in women than in men, as are their associated pulmonary manifestations. However, certain conditions, such as rheumatoid arthritis, may lead to a higher likelihood of respiratory complications in men compared to women [2, 3].

In the ICU setting, ILD symptoms may worsen, often accompanied by signs of ARDS, such as severe hypoxemia and diffuse pulmonary infiltrates on imaging. Blood tests and serum chemistries are generally non-specific and provide limited diagnostic value. However, testing for rheumatic diseases or vasculitis using specific antibodies, such as antinuclear antibodies (ANAs), rheumatoid factor (RF), erythrocyte sedimentation rate (ESR), C-reactive protein (CRP), anti-citrullinated peptide antibody (ACPA), anti-neutrophil cytoplasmic antibody (ANCA), and anti-glomerular basement membrane (anti-GBM) antibodies, may be helpful in some cases. Serum precipitins can also identify common hypersensitivity antigens. While the angiotensin-converting enzyme (ACE) test is more sensitive than specific, it may provide diagnostic clues for sarcoidosis [4, 5]. High-resolution computed tomography (HRCT) is the most effective noninvasive method for diagnosing ILD, offering detailed imaging of lung parenchymal abnormalities. Findings on high-resolution computed tomography (HRCT) typically include ground-glass opacities. Less common patterns include cryptogenic organizing pneumonia (COP), nodules, pulmonary Langerhans cell histiocytosis, and respiratory bronchiolitis. Large cystic spaces may be observed in PLCH and lymphangioleiomyomatosis (LAM), while honeycombing indicates end-stage disease and is associated with a poor prognosis. Lung biopsy and bronchoalveolar lavage can provide tissue samples for histopathological examination, though these procedures carry higher risks in critically ill patients. Pulmonary function tests often reveal reduced lung volumes, including total lung capacity (TLC), forced expiratory volume in one second (FEV1), and forced vital capacity (FVC). The FEV1/FVC ratio is typically normal or increased, while the diffusing capacity for carbon monoxide (DLCO) is generally reduced. Static pulmonary compliance decreases progressively in correlation with the extent of fibrosis. Bronchoalveolar lavage findings are often abnormal but usually nondiagnostic, though they can help evaluate for infection or malignancy and diagnose eosinophilic pneumonia. Transbronchial and endobronchial lung biopsies may be diagnostic for sarcoidosis or lymphangitic spread of carcinoma but are often nondiagnostic for other diseases due to their patchy distribution. Many patients require open or thoracoscopic lung biopsy for a definitive diagnosis, with video-assisted thoracoscopic lung biopsy being the preferred method. The role of lung biopsy in the context of characteristic HRCT findings remains controversial, with expert opinions divided. However, consensus is growing toward avoiding lung biopsy when typical clinical and HRCT features of usual interstitial pneumonia (UIP) or idiopathic pulmonary fibrosis (IPF) are present [7, 8]. Acute exacerbation of interstitial lung disease (AE-ILD) is characterized by the abrupt worsening of ILD-related symptoms, including dyspnea, cough, and increased work of breathing. These exacerbations are often precipitated by infections, such as viral or bacterial pathogens, and environmental triggers like pollution may also play a role. Underlying disease progression is one of the most challenging triggers of AE-ILD, as treatment options are typically limited, and the likelihood of recovery is low. The natural history of diffuse interstitial lung diseases varies among diagnostic entities and individuals with the same diagnosis. Some diseases have an insidious onset and progress gradually but relentlessly, while others present acutely but respond well to therapy. This comprehensive literature review was conducted to establish a framework for understanding disease progression and management strategies in patients with ILD. For intensivists, managing acute exacerbations of ILD (AE-ILD) is a high-stakes balancing act. These patients often arrive in the ICU with severe respiratory failure, but distinguishing AE-ILD from pneumonia, heart failure, or ARDS can be challenging. Clinical and imaging features overlap widely—ground-glass opacities on a CT scan, for example, could signal AE-ILD *or* ARDS—yet the treatment paths for these conditions differ starkly. Compounding this uncertainty are the unique physiological hurdles posed by fibrotic lungs: stiff, scarred tissue resists recruitment, making mechanical ventilation a delicate tightrope walk between sustaining oxygenation and avoiding further harm. With mortality rates exceeding 50% in many studies, the stakes could not be higher. This urgency demands not only sharper diagnostic tools but also clearer prognostic markers to guide decisions about aggressive interventions versus palliative care.

This review responds to these challenges by synthesizing the evidence on managing AE-ILD in critical care, and exploring how tailored ventilator strategies, antifibrotic therapies, and advanced support like ECMO might improve outcomes for these vulnerable patients. Beyond treatment, factors that could help clinicians predict survival, such as the severity of hypoxemia, ventilatory ratios, or patterns on imaging were highlighted

## Methodology

### Databases

A systematic search was conducted in reputable databases, including PubMed, Google Scholar, and Embase. To ensure a comprehensive search, a combination of keywords such as “interstitial lung disease,” “ILD,” “intensive care,” and “outcomes” was used.

### Inclusion and Exclusion Criteria

Studies published within the last ten years reporting on the outcomes of ILD patients admitted to intensive care were included. Studies focusing on pediatric populations or non-human subjects were excluded. Eligibility criteria included observational studies, clinical trials, and systematic reviews that report the outcomes of ILD patients admitted to the ICU. Studies focusing on prognostic factors, management strategies, and palliative care interventions were also included.

### Data Extraction

Relevant data on patient demographics, clinical characteristics, interventions, and outcomes were reviewed, extracted, and synthesized to provide a comprehensive literature overview.

### Data Synthesis and Analysis

The findings from selected studies were summarized, emphasizing key outcomes and factors influencing them.

### Ethical Considerations

Ethical guidelines and principles were strictly followed throughout the review process, ensuring responsible and respectful handling of data and information.

## Results

### General Management Principles

Management of ILD in the ICU includes disease-specific treatments and supportive care measures. The primary goals are to manage the underlying cause of lung disease, relieve symptoms, and prevent further complications. Specific treatment strategies, such as immunosuppressive therapy (e.g., corticosteroids, azathioprine, or mycophenolate mofetil), are commonly used in ILD associated with autoimmune conditions. Antifibrotic agents, such as pirfenidone and nintedanib, are approved for the treatment of idiopathic pulmonary fibrosis and can help slow disease progression. Patients experiencing severe respiratory failure may require mechanical ventilation. However, the use of mechanical ventilation requires careful consideration due to the risk of ventilator-induced lung injury. Supplemental oxygen and pulmonary rehabilitation can provide additional support. In the context of IPF, If microbiological culture data are unavailable, clinical judgment and empirical treatment based on the patient’s presentation and risk factors should guide management. Additionally, many patients with non-IPF ILD undergoing long-term immunosuppressive treatment are susceptible to opportunistic infections, bronchoalveolar lavage (BAL) should be carefully evaluated as part of the diagnostic approach in cases of acute exacerbation of ILD, alongside blood cultures, Legionella and pneumococcal urine antigen testing, serum galactomannan levels, cryptococcus antigen testing, and assessment for endemic fungi. Patients diagnosed with connective tissue disease-related ILD should be thoroughly evaluated for other potential contributors to acute respiratory failure, including drug toxicity and diffuse alveolar hemorrhage (DAH), in addition to infection. Drug-induced pneumonitis can arise from medications used in the treatment of connective tissue diseases (CTD), such as methotrexate, leflunomide, sulfasalazine, tumor necrosis factor-alpha inhibitors, and cyclophosphamide. DAH, often caused by minor vessel inflammation, is more common in patients with lupus and less frequently in those with rheumatoid arthritis, mixed connective tissue disease, systemic sclerosis, and dermatomyositis. DAH can be detected by analyzing sequential BAL samples, which show a progressive increase in red blood cell (RBC) count. Patients diagnosed with anti-MDA5 dermatomyositis face a high risk of rapidly progressive ILD, which can lead to acute respiratory distress syndrome (ARDS) [9,10,11].

### Management of ILD in the ICU

A previous study found that most patients with IPF (93%) were hospitalized during the last six months of their lives, and 80% of enrolled patients died in the hospital [12]. Clinical data indicate that noninvasive ventilation (NIV) yields better outcomes than invasive mechanical ventilation (IMV) in ILD patients. One study reported a mortality rate of 35% among NIV patients, compared to 100% for those treated with IMV [13]. Mechanical ventilation is often required for severe respiratory failure in ILD patients. However, the prognosis for ILD patients requiring IMV is generally poor. Studies have shown that approximately 70% of Patients diagnosed with ILD who require invasive mechanical ventilation have a high mortality rate. For example, Fernández-Pérez et al. (2008) reported a mortality rate of 78% among ventilated ILD patients. Chronic ILD patients exhibit physiological changes that complicate IMV during acute exacerbations. IPF, characterized by decreased lung compliance due to alterations in the extracellular matrix and surfactant, may lead to cyclic opening and closing of alveolar units during tidal breathing. It remains unclear whether these units can be recruited using positive end-expiratory pressure (PEEP). Low lung compliance and poor alveolar recruitment increase susceptibility to ventilator-induced lung injury [9, 13, 14]. Historically, the prognosis for ILD patients treated with IMV has been poor, particularly if lung transplantation (LTx) is not an option. A recent national multicenter study revealed that 78% of patients requiring IMV for acute exacerbation of ILD died within 30 days without undergoing LTx, and this number rose to 96% within six months. Only 5% of IPF patients survived hospitalization after requiring IMV for acute exacerbation of ILD (AE-ILD), compared to 14% of patients with connective tissue disease-associated ILD. Two large population-based studies using the US National Inpatient Sample found that 11% of IPF patients admitted to hospitals underwent IMV, with mortality rates ranging from 49% to 56%, and only 10% were discharged alive. It remains uncertain whether AE-ILD or other factors directly necessitated mechanical ventilation. Predictors of mortality among AE-ILD patients receiving IMV include a low ratio of arterial oxygen partial pressure to fractional inspired oxygen PaO_2_/FiO_2_ (<100 mm Hg), elevated carbon dioxide levels, high PEEP and plateau pressures, and disease progression as the indication for mechanical ventilation. Limited research on ventilator strategies for AEILD suggests that PEEP may be less effective in ILD patients due to reduced lung compliance and alveolar recruitability. One study found that PEEP levels above 10 cm H_2_O increased plateau pressure and reduced driving pressure and compliance without improving PaO_2_/FiO_2_. A prospective trial involving five patients (one with IPF and four with CTD-ILD) demonstrated that prone positioning reduced plateau pressure and compliance without altering the PaO_2_/FiO_2_ ratio [9]. NIV has been shown to reduce intubation rates and inhospital mortality for patients with acute hypoxemic respiratory failure, according to a meta-analysis of randomized controlled trials. The primary physiological benefit of PEEP is improved oxygenation through enhanced alveolar recruitment. Bi-level non-invasive ventilation (BPAP-NIPPV) can reduce respiratory distress and lower carbon dioxide (CO_2_) levels. Although hypercapnia is uncommon in stable ILD patients, it may occur due to weakened respiratory muscles or reduced lung compliance with higher mechanical power, making NIV beneficial for AE-ILD. However, concerns about high PEEP levels may limit its use in this setting [15]. Retrospective studies on NIV for AE-ILD have shown that nearly half of the patients who received NIV avoided intubation. A higher respiratory rate predicted NIV failure, while age, FVC, and radiographic extent of ILD did not. However, the median survival for patients with “NIV success” was only 90 days, highlighting the poor outcomes of AE-ILD even for those who survive hospitalization. A multicenter retrospective study found that 18% of patients developed intolerance to the mask interface/pressure after a median of 72 hours of NIV use. While the PaO_2_/FiO_2_ ratio improved significantly after six hours of NIV, this improvement was only observed in patients with pneumonia. Therefore, the cause of acute exacerbations or worsening hypoxemia should be considered when selecting supportive modalities [16,17,18]. High-flow nasal cannula (HFNC) therapy is a viable respiratory support option for ILD patients. HFNC delivers heated and humidified oxygen at high flow rates, reducing breathing effort and improving oxygenation. Some ILD patients may benefit from HFNC therapy, potentially reducing the need for intubation. Further research is needed to determine its effectiveness and best practices in this population. Compared to standard oxygen therapy, HFNC offers physiological benefits such as reduced respiratory secretions, decreased dead space, provision of low-level PEEP, and lower respiratory rate and work of breathing. A small retrospective analysis found that AE-ILD patients treated with HFNC had a lower risk of intubation and a 30-day mortality rate of 23%, compared to 63% for those treated with NIV. HFNC was the only significant independent predictor of 30-day survival, with a hazard ratio of 0.15 (95% confidence interval: 0.03–0.88). These differences may be attributed to unmeasured confounders, as the choice of respiratory support device was based on clinician judgment [19]. Extracorporeal membrane oxygenation (ECMO) shows promise as a treatment for ILD patients with acute respiratory failure, particularly those eligible for lung transplantation. However, for non-transplant candidates, ECMO does not significantly improve survival outcomes [20]. Guidelines recommend lung transplantation for ILD patients who meet established criteria. While most lung transplants for ILD patients are performed on an outpatient basis for those with stable but progressive disease, a small subset of patients undergo transplantation due to acute exacerbation of IPF. A single-center study of 89 IPF patients who underwent lung transplantation found that 37 experienced acute exacerbation of ILD before being listed for transplantation. Of these, 28 underwent successful transplantation, with a 30-day posttransplant survival rate of 93% (compared to 100% for stable IPF patients). However, AE-ILD patients had significantly lower 1- and 2-year survival rates than stable IPF patients. Among AE-ILD patients, 33% required ECMO support, and only 33% of these survived to transplantation [21]. Data from the United Network for Organ Sharing indicate that 74% of patients who received ECMO as a bridge to transplant (BTT) had ILD. Of these, 62% underwent lung transplantation, 16% died before transplantation, 15% were removed from the waitlist due to disease progression, and only 1% survived hospitalization without transplantation [22]. An initial single-center study of ECMO as a bridge to transplantation in ILD patients reported a 1-year survival rate of 27%. More recent studies have shown improved 1-year survival rates of 56% and 74% with ECMO as a bridge to transplantation [23]. ECMO has been used in patients with anti-MDA5 dermatomyositis. Among 15 anti-MDA5 patients with rapidly progressive ILD who received ECMO, five underwent lung transplantation, while the remaining ten died after a median of 30 days on ECMO. This highlights the limited utility of ECMO as a “bridge to recovery” for non-transplant candidates with acute exacerbation of ILD, whether related to anti-MDA5 dermatomyositis or other ILD types. Contraindications for lung transplantation in ILD patients may include age >65 years, BMI >35 or <16 kg/m^2^, severe coronary artery disease, lack of stable social support, severe esophageal motility disorder, or significant psychological illness [24, 25].

### Predictive Variables Associated with Poor Outcomes in ICU Settings

**Fig. 1.**
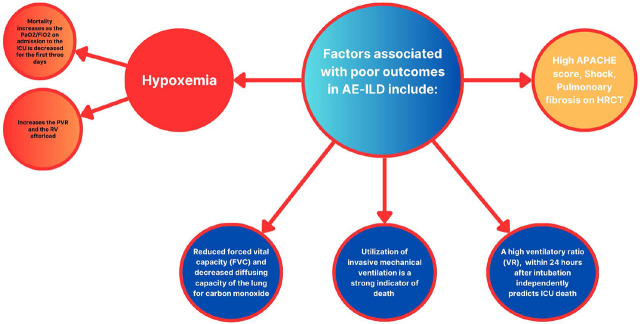
Factors associated with poor outcomes in AE-ILD

Patients admitted to the ICU with ILD are influenced by various factors that affect their prognosis (Figure 1). Disease severity at admission, reflecting the extent of lung fibrosis and respiratory failure, is a significant determinant of mortality. Poor outcomes are typically observed in patients with advanced fibrotic disease and pronounced hypoxemia. Hypoxemia severity, particularly in IPF and mixed-ILD, is strongly associated with mortality, especially when the PaO_2_/FiO_2_ ratio is low during the first three days of ICU admission [14, 26]. Impaired lung function, particularly reduced forced vital capacity (FVC) and diffusing capacity for carbon monoxide (DLCO), increases the risk of acute exacerbation of ILD (AE-ILD). The underlying ILD type also predicts ICU outcomes, with IPF having the highest mortality rate among ILDs, though this has decreased over time [14]. In mixed-ILD patients, higher Acute Physiology and Chronic Health Evaluation (APACHE) scores are associated with increased mortality [14, 27]. Protective factors include a higher oxygenation index and conventional oxygen therapy, while shock, pulmonary fibrosis on CT scans, and NIV use are poor prognostic factors [28]. Acute exacerbations of ILD, characterized by abrupt deterioration of respiratory symptoms and lung function, are associated with high mortality rates. For example, acute exacerbations of IPF have mortality rates exceeding 50%. A single-center retrospective study of 220 patients found that 20% of acute exacerbations were caused by infection [29]. Comorbidities such as cardiovascular disease, diabetes, and chronic kidney disease negatively impact outcomes. Older age, worse functional status, frailty, and diminished physical reserve are also associated with higher ICU mortality [29]. The need for IMV is a strong predictor of mortality in ILD patients, with studies consistently showing higher death rates among those requiring ventilatory support. High PEEP settings are also associated with lower survival rates [14, 27]. A high ventilatory ratio (VR), calculated as VR = (V(E measured) × Pa(CO_2_ measured))/(V(E predicted) × Pa (CO_2_ predicted)) within 24 hours of intubation independently predicts ICU mortality [30, 31]. A study found that the primary causes of acute respiratory failure were worsening IPF and pneumonia, with overall hospital mortality rates of 43.8%, rising to 56.7% for patients requiring IMV. The mortality rate for IMV in IPF exacerbation was 81.3% [32]. The annual incidence of AE-IPF ranges from 4% to 20%, with hospital mortality rates exceeding 50% and ICU mortality rates approaching 90%. Median survival after AE-IPF ranges from 15.5 to 36 months, significantly shorter than for IPF patients without acute exacerbation [32]. Non-IPF ILD patients have a lower risk of acute exacerbation than IPF patients, with connective tissue disease-associated ILD patients experiencing slightly better outcomes. However, mortality rates remain high [33]. Comorbidities such as cardiovascular disease, pulmonary hypertension, diabetes, and chronic kidney disease further worsen outcomes in ILD patients [34, 35, 36]. Long-term prognosis after ICU admission is poor, with a 2-year survival rate of only 36% among ILD patients [37]. Multi-organ failure and acute exacerbation of ILD are strongly associated with in-hospital mortality, with odds ratios of 12.6 and 5.4, respectively [37]. A study of 145 patients with diffuse parenchymal lung disease (DPLD) admitted to the ICU for acute respiratory failure found an overall mortality rate of 45.5%, with the highest rates in acute interstitial pneumonia (82%) and IPF (59%) [38]. A single-center retrospective study of 126 ILD patients admitted to the ICU reported hospital and 1-year mortality rates of 66% and 80%, respectively. Connective tissue disease-associated ILD patients had lower short- and long-term mortality rates than those with unclassifiable ILD [39]. Another retrospective study of 72 ILD patients found in-hospital mortality rates of 68% for IPF, 40% for drug-induced ILD, and 25% for other ILD types, rising to 100%, 64%, and 60% for patients requiring IMV [40]. A multicenter study of systemic rheumatic disease (SRD) patients with ILD found significantly higher mortality rates compared to SRD patients without ILD [41]. The median ICU length of stay for ILD patients is approximately 14 days, with average costs exceeding $100,000 per admission [42, 43]. Pulmonary rehabilitation can improve functional outcomes and quality of life in ILD patients, though the degree of improvement varies [44]. Health-related quality of life (HRQoL) is significantly reduced after ICU admission, with chronic symptoms, psychological distress, and long-term oxygen therapy contributing to poor outcomes [45]. Psychological support is essential for addressing depression, anxiety, and post-traumatic stress disorder (PTSD) in ICU survivors [46].

## Discussion

The pathophysiology of AE-ILD involves the activation of an inflammatory cascade, including the release of pro-inflammatory cytokines and alveolar inflammatory cellular infiltration. This process leads to further progression of diffuse alveolar damage, thickening of the alveolar-capillary membrane, increased pulmonary vascular resistance, and impaired blood perfusion. These changes collectively impair the lung’s gas exchange capability, resulting in varying degrees of hypoxemia and hypercarbia [47, 48]. ICU survival outcomes for patients with interstitial lung disease (ILD) vary widely and are influenced by the patient’s baseline health, the status and progression of ILD, and the underlying etiology of the disease. ICU care is typically necessitated by acute respiratory failure, a common complication of ILD and the primary cause of ICU admission. Severe and progressive respiratory failure is associated with a limited likelihood of successful treatment and complete recovery [49]. Critical care physicians often face severe exacerbations that carry significant risks. These acute events occur suddenly, leading to rapid deterioration of pulmonary function and necessitating ventilatory support in the ICU. Many studies highlight that patients with fibrotic ILD, particularly idiopathic pulmonary fibrosis (IPF), have a low chance of recovery when admitted to the ICU. For instance, a cohort study demonstrated that many patients did not survive beyond their ICU stay after requiring invasive mechanical ventilation (IMV). Ventilating these patients can be challenging due to mechanical and physiological factors, as described by the “squishy ball lung” theory. In this model, fibrotic lung regions act as rigid structures, leading to uneven air distribution and increasing the risk of ventilator-induced lung injury (VILI) [50]. The most effective strategies for managing oxygenation and ventilation in ILD patients in the ICU must balance the need for adequate gas exchange with the risks of further lung injury. Low tidal volume ventilation is a standard recommendation to reduce barotrauma and volume trauma. However, care must be taken to avoid exacerbating lung damage. In the meantime, principles derived from ARDS management should guide current practice. This is particularly challenging when managing patients with non-invasive mechanical ventilation (NIV), as tidal volume cannot be controlled. A high respiratory drive in these patients may increase lung stress and mechanical power, leading to patient-induced self-lung injury or ventilation-induced lung injury.

AE-ILD can be managed pharmacologically using corticosteroids, antifibrotic agents, or immunosuppressants, among other drugs. However, even with these interventions, the prognosis remains guarded. Although nintedanib and pirfenidone are effective in slowing disease progression in chronic ILD, there is limited evidence supporting their use during acute exacerbations. The use of corticosteroids has not consistently improved survival rates, though they may help reduce inflammation in some cases. Physicians should identify prognostic factors when treating ILD patients in the ICU, as these may provide insights into the most effective management strategies. Factors such as the extent of fibrosis on CT scans, the presence of pulmonary hypertension, and the need for IMV are often associated with worse outcomes. A usual interstitial pneumonia pattern on CT scans is particularly indicative of a poor prognosis compared to other ILD types [51]. The complex nature and pathology of ILDs significantly impact ICU outcomes. Currently, there is no established criterion for ICU admission specific to ILD patients beyond the physiological criteria used for typical cases. Factors such as the type of ILD, age, comorbidities, invasiveness of treatment strategies, and overall clinical condition must be considered when selecting treatment methods for individual patients [11, 39]. Pulmonary hypertension (PH) complicating interstitial lung disease (ILD) is associated with increased mortality due to hypoxia-induced vascular remodeling, fibrosis-related capillary loss, and elevated pulmonary vascular resistance. In critically ill ILD patients, diagnosing PH is challenging due to overlapping clinical features with ILD mainly in terms of dyspnea and hypoxemia. limitations of echocardiography in fibrotic or hyperinflated lungs, and the procedural risks of right heart catheterization in hemodynamically unstable patients [52,53]. Management in the ICU requires careful consideration of competing risks. Oxygen therapy and diuretics remain foundational to alleviate hypoxemia and volume overload, respectively. However, pulmonary vasodilators such as sildenafil are used cautiously due to inconsistent evidence and potential exacerbation of ventilation-perfusion mismatching in fibrotic lung regions. Mechanical ventilation strategies, such as lung protective tidal volumes and judicious PEEP, must balance alveolar recruitment with hemodynamic stability in the context of right ventricular dysfunction. Extracorporeal membrane oxygenation (ECMO) is rarely beneficial, with only 22% of PH-ILD patients surviving to lung transplantation [54,55]. Given the guarded prognosis (median survival <1 year), early integration of palliative care is critical to address refractory dyspnea, clarify goals of care, and prioritize patient-centered outcomes [56,57].

## Conclusion

This literature review points out outcome predictors of interstitial lung disease in intensive care units, which are mainly hypoxemia, the severity of the illness, invasive ventilation, the presence of shock, and the extent of fibrosis on CT Images.
